# Indeterminate and oncogenic potential: CHIP vs CHOP mutations in AML with *NPM1* alteration

**DOI:** 10.1038/s41375-021-01368-1

**Published:** 2021-08-10

**Authors:** Luca Vincenzo Cappelli, Manja Meggendorfer, Constance Baer, Niroshan Nadarajah, Stephan Hutter, Sabine Jeromin, Frank Dicker, Wolfgang Kern, Torsten Haferlach, Claudia Haferlach, Alexander Höllein

**Affiliations:** 1 MLL Munich Leukemia Laboratory, Munich, Germany; 2 Dept. of Translational and Precision Medicine, Sapienza University, Rome, Italy; 3 Present Address: Department of Hematology and Oncology, Rotkreuzklinikum München, Munich, Germany

**Keywords:** Cancer genetics, Cancer genetics

## Abstract

In AML patients, recurrent mutations were shown to persist in remission, however, only some have a prognostic value and persistent mutations might therefore reflect a re-established premalignant state or truly active disease causing relapse. We aimed to dissect the nature of co-mutations in *NPM1* mutated AML where the detection of *NPM1* transcripts allows highly specific and sensitive detection of complete molecular remission (CMR). We analysed 150 consecutive patients who achieved CMR following intensive treatment by next generation sequencing on paired samples at diagnosis, CMR and relapse (38/150 patients). Patients with persistence or the acquisition of non-DTA (*DNMT3A*, *TET2*, *ASXL1*) mutations at CMR (23/150 patients, 15%) have a significantly worse prognosis (EFS HR = 2.7, *p* = 0.003; OS HR = 3.6, *p* = 0.012). Based on clonal evolution analysis of diagnostic, CMR and relapse samples, we redefine pre-malignant mutations and include *IDH1*, *IDH2* and *SRSF2* with the DTA genes in this newly defined group. Only the persistence or acquisition of CHOP-like (clonal hematopoiesis of oncogenic potential) mutations was significantly associated with an inferior outcome (EFS HR = 4.5, *p* = 0.0002; OS HR = 5.5, *p* = 0.002). Moreover, the detection of CHOP-like mutations at relapse was detrimental (HR = 4.5, *p* = 0.01). We confirmed these findings in a second independent whole genome sequencing cohort.

## Introduction

Acute myeloid leukemia (AML) is characterized by recurrent genetic aberrations including gene mutations [1]. Using modern next generation sequencing (NGS) techniques, typical recurrent mutations can be detected in up to 90% of all AML patients [2]. Moreover, certain aberrations in genes such as *NPM1* can be exploited to detect minimal residual disease (MRD) with a high sensitivity of up to 1 in 10^6^ cells [3]. The increasing availability and sensitivity of NGS applications have driven attempts to further identify molecular markers for MRD detection. Several large studies have shown persistent mutations at morphologic and clinical remission following intensive treatment of AML [4–7]. This raised the hypothesis that these mutations might reflect active leukemia and thus the presence of minimal residual disease. This notion was underscored when the persistence of mutations at remission was associated with relapse [6]. However, it was shown that not all genes have the same impact on prognosis: the persistence at remission of certain mutations in genes such as *DNMT3A*, *TET2* or *ASXL1* (DTA) was not associated with a worse outcome [4]. What is more, DTA mutations are also the most prevalent gene mutations defining age related clonal hematopoiesis (ARCH [6, 8, 9]) or clonal hematopoiesis of indeterminate potential (CHIP [10]). ARCH has been characterized as a molecular risk factor for the development of hematopoietic disorders including leukemia [11]. However, the presence of certain mutations in otherwise healthy subjects only confers a low risk for transformation [8, 9]. Effort has been put into the discrimination of CHIP-like mutations and mutations that are associated with oncogenic potential (clonal hematopoiesis of oncogenic potential - CHOP) [12]. Therefore, in some cases, the persistence of mutations at remission could reflect the re-establishment of a pre-leukemic state following induction therapy for AML which might not necessitate further treatment. In other cases, the persistence of malignant mutations at remission could truly reflect active disease and therefore warrant intensified treatment strategies [11]. In this light, the existence of harmless CHIP-like and true driver mutations can be hypothesized. To investigate this hypothesis, we analysed paired samples at diagnosis, CMR and relapse of AML patients with mutated *NPM1* (*NPM1*^mut^).

## Methods

### Patients and study design

We performed a retrospective cohort study investigating the prevalence and the spectrum of mutations at diagnosis, CMR and relapse of 150 patients diagnosed with *NPM1*^mut^ AML between 2005 and 2016 at our institution (cohort 1). Diagnosis was assessed by cytomorphology, immunophenotyping and genetic studies according to WHO criteria. Only patients with de novo AML were considered. We included all patients who achieved CMR definded by the absence of *NPM1* transcripts (qPCR ratio 0, sensitivity 0.001%) and excluded patients with a *NPM1* negative relapse. An additional cohort of 36 *NPM1*^mut^ AML patients from the 5000 Genome Project (MLL [13, 14]) was studied by whole genome sequencing (WGS) at diagnosis, CMR and for eight patients at relapse (cohort 2). All patients gave their written informed consent for scientific evaluations. The study was approved by the Internal Review Board and adhered to the tenets of the Declaration of Helsinki. All patients were treated with intensive chemotherapy regimens according to AML standard therapy. The median follow-up of the two cohorts was 3.3 years (range: 0.2–8.7).

### Genetic analyses

For all patients the mutational status of *NPM1* was studied at diagnosis both by melting curve analysis and NGS. All diagnostic, CMR and relapse samples were studied by NGS with a panel of 63 genes associated with hematological malignancies (Supplementary Methods, online only). Library preparation and variants analysis were performed as previously described ([15] and Supplementary Methods). *FLT3*-ITD was analysed by gene scan in all patients. Chromosome banding analysis (CBA) was performed according to standard procedures in all patients. For cohort 2, WGS analysis was performed on all diagnostic, CMR and relapse samples as previously described ([14] and Supplementary Methods). We focused our analyses on the protein-coding regions of the genome. We also took advantage of the multiplicity of samples per patient to filter out those variants bearing a VAF close to 50% or 100% across all timepoints, indicating either heterozygous or homozygous germline variants.

## Results

### Patients’ clinical and molecular characteristics

Between 2005 and 2016, 150 patients with *NPM1*^mut^ AML who achieved a CMR following intensive treatment were included in this study (for details see Table 1 and Supplementary Table 1, online only). Relapse was diagnosed in 34% of patients (52/150), which is in line with previous reports on *NPM1*^mut^ AML following CMR [16]. A total of 61/150 patients (41%) received allogeneic hematopoietic stem-cell transplant (allo-HSCT) up-front (*n* = 34/61, 56%) or after relapse (*n* = 27/61, 44%) with a median time from diagnosis to transplant of 0.8 years (range: 0.5–1.1). In all these patients, the CMR sample was collected prior to HSCT.

**Table 1:** Patients’ clinical and molecular features.

	*n*	%
Gender		
Male	73	49%
Female	77	51%
WHO AML subtype		
AML with minimal differentiation	1	1%
AML without maturation	66	44%
AML with maturation	40	27%
Acute myelomonocytic leukemia	30	20%
Acute monoblastic/monocytic leukemia	8	5%
Pure erythroid leukemia	2	1%
NA	3	2%
ELN risk classification		
Favorable (no *FLT3*-ITD, or *FLT3*-ITD+, ratio <0.5)	116	77%
Intermediate (*FLT3*-IDT+, ratio >0.5)	31	21%
NA	3	2%
Karyotype		
Normal	134	89%
Aberrant^a^	14	9%
NA	2	1%
	Median	Range
Age	57	19–82
Hb	9	4–16
Thrombocytes (x10^3^)	64	7–289
Leukocytes (x10^3^)	30	1–224

*NA* not analyzed.

^a^3/14 (21%): X/Y loss; 1/14 (7%): del(5q); 2/14 (14%): del(9q); 1/14 (7%): ins(10;4); 1/14 (7%): t(3;10); 1/14 (7%): +21; 1/14 (7%): der(1)t(1;13); 3/14 (21%): +8; 1/14 (7%): complex karyotype.

### In *NPM1*^mut^ AML co-mutations persist at CMR

**Fig. 1:**
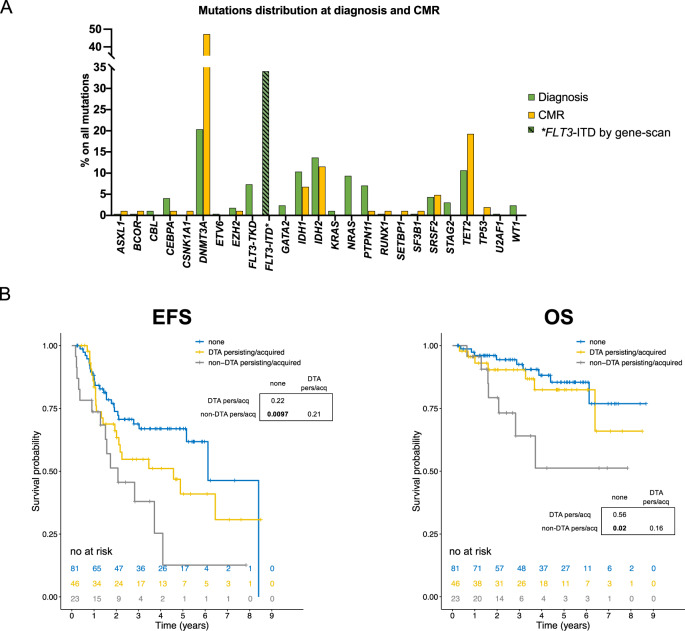
Persisting/acquired non-DTA mutations at CMR confer inferior survival in AML with mutated *NPM1*. **A** Mutation frequencies of AML associated genes in diagnostic and complete molecular remission (CMR) samples. The percentage of each gene alteration among all the mutations per timepoint is depicted. *FLT3-ITD mutations were detected by gene scan. **B** Survival analysis of patients with *NPM1*^mut^ AML stratified by persistence or acquisition of DTA vs non-DTA mutations at CMR. Kaplan–Meier plots depicting event-free survival (EFS left panel) and overall survival (OS right panel) of *NPM1*^mut^ AML patients based on the combination of persistency and acquisition of non-DTA mutations at CMR. Patients showing non-DTA hits at CMR have a worse prognosis than those who do not. *P* values were calculated with the log-rank test and *p* values for pairwise comparisons are given.

At diagnosis, a total of 301 mutations were detected across all 150 patients, excluding *NPM1* (2.1 mutations/patient). Of these, the most common were found in *DTA* genes *DNMT3A* (20%) and *TET2* (11%) (with the exception of *ASXL1* mutations, which is expected given their low frequency in *NPM1*^mut^ AML [16, 17]), plus others including *IDH2* (14%), *IDH1* (10%), *NRAS* (9%), *FLT3-TKD* (7%), *PTPN11* (7%), *SRSF2* (4%), and *CEBPA* (4%) (Fig. 1A). *FLT3*-ITD was identified in 51/150 patients (34%).

At CMR, 69/150 patients carried at least one mutation (46%), using a VAF cutoff of ≥1%, a total of 105 mutations were detected across all 150 patients (0.7 mutations/patient) (Fig. 1A).

This shows that also in *NPM1*^mut^ AML there is an important fraction of patients displaying mutations at remission, either reflecting MRD positivity or CHIP-like premalignant mutations. No effect of *FLT3*-ITD on the probability of persistency/acquisition of mutations at CMR was observed (Supplementary Table 2, online only).

### Persistence and acquisition of non-DTA mutations at CMR can predict the outcome of *NPM1*^mut^ AML

Previous work identified that persisting non-DTA mutations at remission are associated with an inferior prognosis [4]. In our cohort 40/150 patients (27%) had persisting DTA mutations and 22/150 (15%) had persisting non-DTA mutations. We confirm that also in the context of *NPM1*^mut^ AML, patients with persisting non-DTA mutations at CMR had a significantly worse EFS (HR = 2.2, 1.2–4.3, *p* = 0.01) and OS (HR = 3.9, 1.54–10, *p* = 0.004) compared to those without persisting mutations (Supplementary Fig. S1A, Supplementary Table 3, online only). We further addressed the acquisition of mutations at remission as a molecular marker for clinical outcome. Patients with at least one novel non-DTA mutation at CMR showed a significantly inferior EFS (HR = 3, 1.3–7.2, *p* = 0.01) but not OS (HR = 2.8, 0.8–9.7, *p* = 0.1, Supplementary Fig. S1B, Supplementary Table 3, online only). Incorporating both into a single model we show that patients with either persistent or acquired non-DTA mutations at CMR (*n* = 23/150, 15%) had a significantly worse prognosis than those who only had persistent/acquired DTA-mutations (*n* = 46, 31%) or none (*n* = 81, 54%) (EFS HR = 2.7, 1.4–5.2, *p* = 0.003; OS HR = 3.6, 1.3–9.8, *p* = 0.012, Fig. 1B, Supplementary Table 3, online only). We did not observe a survival disadvantage in patients with exclusively persistent *DNMT3A*-R882 or *IDH1/2* mutations at CMR (Supplementary Figs S2A, S2B, online only). Also no impact was observed for ELN risk groups (Supplementary Fig. S2C online only). In a multivariate analysis incorporating allogeneic stem-cell transplantation, aberrant karyotype, gender and age [18] (Supplementary Table 4, online only), the persistency/acquisition of non-DTA mutations at CMR was an independent predictor of outcome (OS HR = 3.8, 1.01–14, *p* = 0.047).

### *NPM1* is a second hit mutation on the basis of underlying CHIP

We have previously shown in a cumulative analysis that comparing the VAF of *NPM1* with co-mutations, *NPM1* was a second hit in the majority of cases [15]. We now analyzed our panel sequencing results for a more accurate assessment of the clonal hierarchy in the diagnostic sample (Supplementary Figs S3A, B online only). As expected, in most of the cases *NPM1* was a second hit mutation, with a VAF lower than co-mutated genes including: *STAG2*, *EZH2*, *DNMT3A*, *IDH1*, *IDH2*, *SRSF2*, and *TET2*.

Moreover, *NPM1* as second hit mutation was age dependent and associated with an increased number of acquired and persistent mutations at CMR (Supplementary Fig. S4, online only). These data suggest that *NPM1* often drives leukemia on the basis of an underlying CHIP.

### Clonal evolution patterns of *NPM1*^mut^ AML from diagnosis to CMR and relapse enables classification of co-mutations

**Fig. 2:**
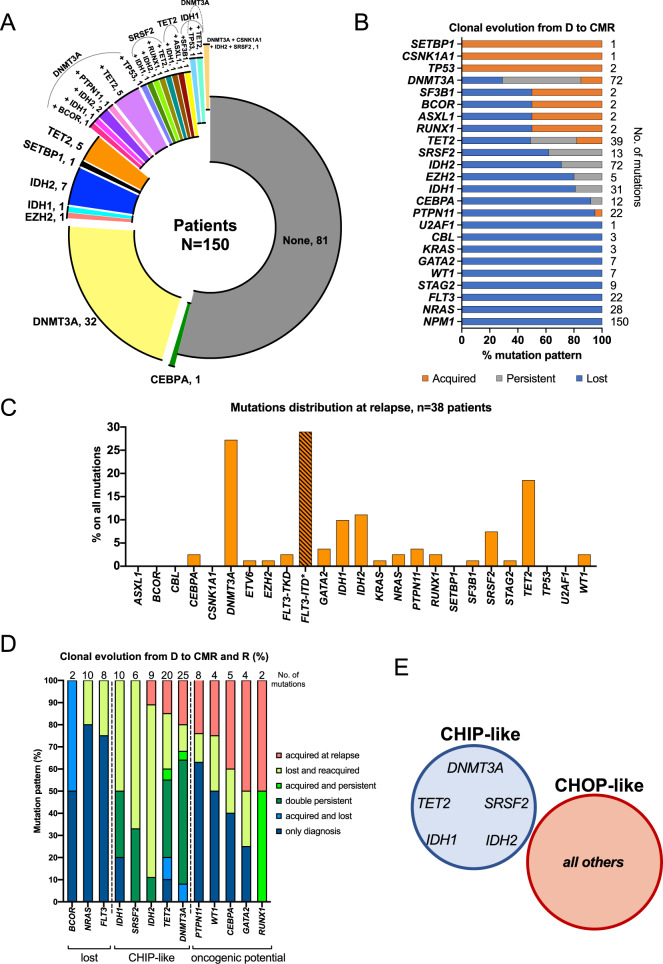
Clonal evolution defines persistent CHIP and CHOP-like mutations in *NPM1*^mut^ AML. **A** Donut plot depicting the mutational status of patients at complete molecular remission (CMR). 81/150 patients (54%) had no mutation, whereas 69/150 (46%) had persistency/acquisition of single or combined mutations, for a total of 24 different groups (donut slices). **B** Clonal evolution analysis of *NPM1*^mut^ AML from diagnosis to CMR (*n* = 150 patients). Lost mutations are depicted in blue, persistent mutations in gray and acquired mutations in orange. Three main patterns emerged: mutations that were mostly or completely lost at CMR: *NRAS*, *FLT3*-TKD, *STAG2*, *WT1*, *GATA2*, and *KRAS* (all 100%), *PTPN11* (95%), *CEBPA* (92%), *IDH1* (81%), *EZH2* (80%), *IDH2* (71%); mutations that were mostly or exclusively acquired at CMR (*TP53*, *CSNK1A1* and *SETBP1*, all 100%), and mutations with a more heterogeneous behavior: *SRSF2* (mutation lost in 45% persistent in 38% and acquired in 17% of cases), *TET2* (mutation lost in 52%, persistent in 31% and acquired in 17% of cases) and *DNMT3A* (mutation lost in 29%, persistent in 56% and acquired in 15% of cases). **C** Mutation frequencies of AML associated genes in relapse samples of 38/52 patients with clinical relapse. The percentage of each gene alteration among all the mutations per timepoint is depicted. *FLT3-ITD mutations were detected by gene scan. **D** Clonal evolution analysis of *NPM1*^mut^ AML from diagnosis to CMR and relapse (R) (*n* = 38 patients) allows for higher temporal resolution and identifies three main patterns: mutations which could either persist at CMR and be lost at R or completely absent at both CMR and R (*BCOR*, *NRAS*, *FLT3*-TKD); CHIP-like mutations present at diagnosis, CMR and R (*TET2*, *IDH1*, *IDH2*, *DNMT3A*, *SRSF2*); mutations with oncogenic potential: gained at CMR and persistent at R or acquired de novo at relapse (*CEBPA, PTPN11, WT1, GATA2*, *RUNX1*). **E** *DNMT3A*, *TET2*, *IDH1*, *IDH2*, and *SRSF2* often act as foundation mutations onto which other potentially oncogenic (CHOP) hits arise as later events in AML pathogenesis. Venn diagram showing the novel proposed classification of CHIP-like mutations including: *DNMT3A, TET2, IDH1, IDH2*, and *SRSF2*, versus mutations with oncogenic potential (CHOP) in the context of *NPM1*^mut^ AML.

In our cohort, 81/150 patients (54%) had no detectable mutation at CMR, whereas 69/150 (46%) showed persistency/acquisition of single or combined mutations, resulting in a total of 24 different groups. Interestingly, persistent *DNMT3A*, *TET2*, and *SRSF2* mutations possibly define subgroups with similar co-mutational patterns (Fig. 2A). By analyzing the clonal evolution of mutations comparing diagnosis and CMR samples, we identified a group of mutations which were completely or mostly lost, a second group which were almost exclusively acquired at CMR, and a third group with more heterogeneous behavior (Fig. 2B).

For 38/52 patients who relapsed, a corresponding sample was available. We detected a total of 84 mutations excluding *NPM1* (2.2 mutations/patient). Significantly more patients had detectable co-mutations at relapse than at diagnosis (100% vs 47%, *p* < 0.0001). As expected, the most common hits were found in *DNMT3A* (27%), *TET2* (19%), *IDH2* (11%), *IDH1* (10%) and *SRSF2* (7%) (Fig. 2C). *FLT3*-ITD was identified in 11/38 patients (29%) by gene scan.

The analysis of 38 relapse samples allowed a higher temporal resolution and a higher degree of differentiation: focusing on those genes that were mutated in at least 2/38 patients, we establish 3 patterns of clonal evolution (Fig. 2D): mutations which were never present at relapse (*BCOR*) or often lost at relapse (*NRAS, FLT3*-TKD); CHIP-like mutations: present at diagnosis, CMR and relapse (*TET2*, *IDH1*, *IDH2*, *DNMT3A*, *SRSF2*); mutations with oncogenic potential: gained at CMR and persistent at relapse or acquired de novo at relapse (*PTPN11*, *WT1, CEBPA*, *GATA2*, *RUNX1*).

### Novel definition of CHIP-like vs CHOP-like mutations provides better prognostic stratification in *NPM1*^mut^ AML

Our analysis shows that next to aberrations in DTA genes, aberrations in *SRSF2*, *IDH2* and *IDH1* could act as CHIP-like mutations in *NPM1*^mut^ AML. This is supported by the clonal hierarchy at diagnosis (Supplementary Figs S3A, B online only). The high number of patient subgroups resulting from the diversity of persistent mutations at CMR (Fig. 2A) abrogates the analysis of their specific outcome. Thus, we implemented a novel classification of CHIP vs CHOP-like mutations in order to identify clinically relevant subpopulations.

**Fig. 3:**
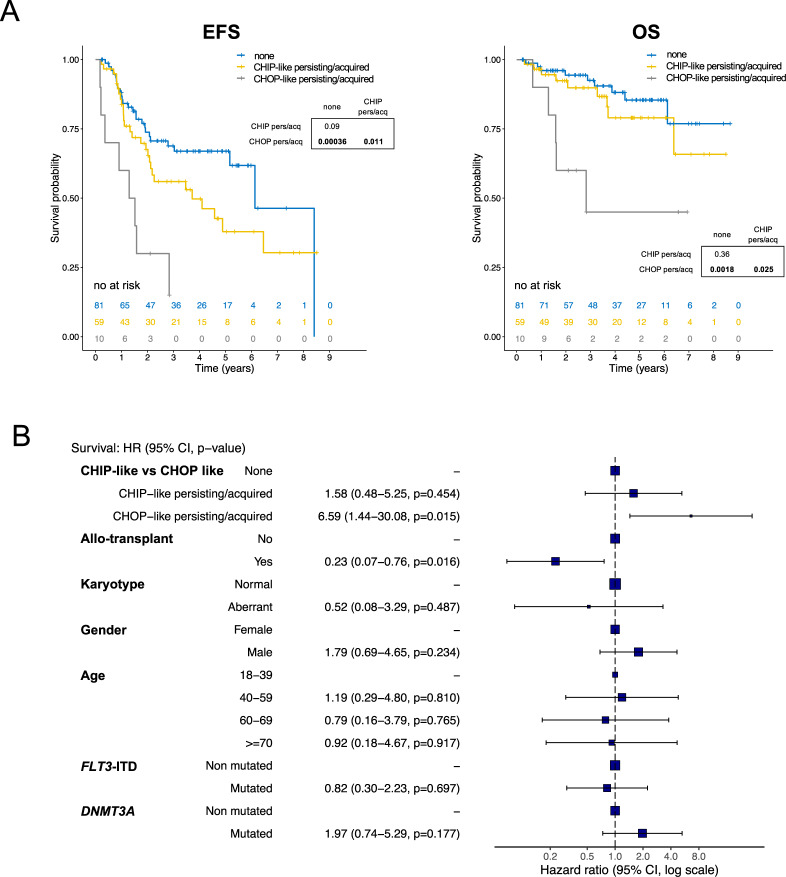
Persistence or acquisition of novel defined premalignant CHIP like mutations vs CHOP like mutations are prognostic in AML with mutated *NPM1*. **A**, **B** Survival analysis of patients with *NPM1*^mut^ AML stratified by clonal evolution patterns of novel defined CHIP-like mutations vs oncogenic mutations. Based on the clonal evolution analysis on diagnosis, remission and relapse samples we redefined CHIP-like mutations (*DNMT3A*, *TET2*, *SRSF2*, *IDH2*, and *IDH1)* versus all other mutations (CHOP-like). **A** Kaplan–Meier plots depicting event-free survival (EFS, left panel and OS, right panel) of *NPM1*^mut^ AML patients based on the persistency/acquisition of CHIP vs CHOP like mutations at CMR. *P* values were calculated with the log-rank test and *p* values for pairwise comparisons are given. **B** Cox proportional hazards multivariate model incorporating clonal evolution patterns by the presence/absence of CHOP-like mutations at CMR, clinical/molecular risk factors and allogeneic hematopoietic stem-cell transplantation. Overall survival (OS) hazard ratio (HR) at 95% confidence interval and *p* values for each variable are given.

We incorporated *DNMT3A*, *TET2*, *IDH1*, *IDH2*, and *SRSF2* in a single category (namely mutations of indeterminate potential, CHIP-like mutations, Fig. 2D and E) and assessed their impact on survival. As a comparator we used all non-CHIP mutations, which we defined as CHOP-like (mutations of oncogenic potential, Fig. 3A, Supplementary Table 5, online only). Interestingly, this led to a stronger predictive power than the restriction on DTA genes alone: 10 patients (7%) with persistence and/or acquisition of CHOP-like mutations had a significantly inferior outcome compared to those who only had CHIP-like persistent/acquired mutations (*n* = 59, 39%) or none (*n* = 81, 54%) (EFS HR = 4.5, 2.0–10.1, *p* = 0.0002; OS HR = 5.5, 1.8–16.9, *p* = 0.002). We did not observe a significant effect on survival when focusing on persistent/acquired *IDH1/2* and *SRSF2* mutations (Supplementary Fig. S5, online only). We finally validated our findings in a multivariate model, incorporating the above-defined factors (Fig. 3B): the persistency/acquisition of CHOP-like mutations at CMR was an independent predictor of outcome (HR = 7, 1.6–30, *p* = 0.009), and was stronger compared to the previous model (Supplementary Table 4, online only) (HR = 7 vs 3.8; log-rank score: 20.5, vs 14.7; *p* value: 0.009 vs 0.04). Of note, the presence of CHIP vs CHOP mutations at CMR was not biased by therapeutic regimens administered (Supplementary Fig. S6, online only). In addition, we observed that the detection of co-mutations at diagnosis (CHIP or CHOP) did not have any impact on OS (Supplementary Fig. S7, online only).

### At relapse persistence/acquisition of CHOP-like mutations identifies high risk patients

**Fig. 4:**
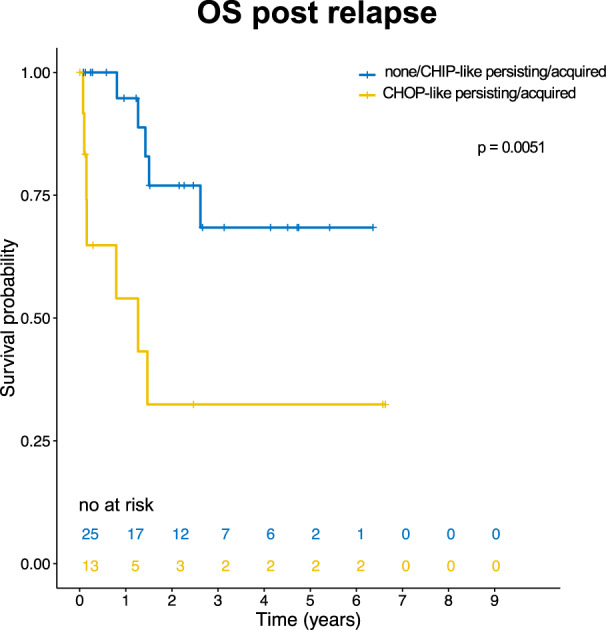
CHOP-like persistent or acquired mutations at relapse confer inferior outcome. Survival analysis of patients with *NPM1*^mut^ AML experiencing clinical relapse stratified by clonal evolution patterns. Kaplan–Meier plots depicting overall survival (OS) of *NPM1*^mut^ AML patients following relapse all analysed by panel sequencing (*n* = 38). We stratified patients that acquired oncogenic mutations at relapse vs patients with no novel or only novel CHIP-like mutations at relapse. OS after relapse was calculated from the date of relapse until the date of death or censoring. *P* values were calculated with the log-rank test.

To our knowledge, our analysis is the first to include mutational screening at relapse in *NPM1*^mut^ AML following CMR. We were able to analyze 38 patients who experienced clinical relapse (Fig. 2C). We focused on the above-defined group of CHIP-like and CHOP-like mutations (Fig. 2E) and show that 13/38 patients (34%) who had persistent/acquired CHOP-like mutations at relapse had a significantly worse outcome following relapse (HR of death after relapse = 4.5, 1.4–14.3, *p* = 0.01, Fig. 4).

### Independent WGS cohort confirms the clinical impact of persisting CHOP-like mutations

We were able to analyze data from an additional 36 *NPM1*^mut^ AML patients which were sequenced both at diagnosis and complete remission by WGS as part of the MLL 5000 genomes project [13, 14]. Eight of them (22%) experienced a clinical and molecular progression (*NPM1*^mut^) and we sequenced the relapse samples by WGS. Clinical characteristics of this cohort are given in Supplementary Table 6 (Supplementary Table 6, online only).

**Fig. 5:**
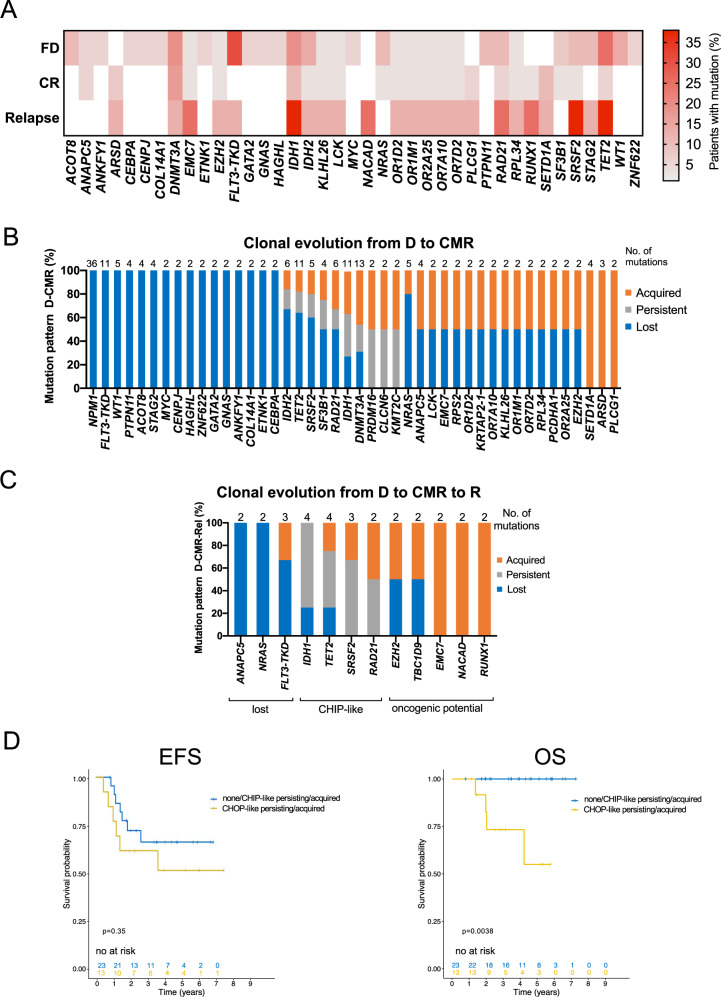
Mutational analysis of independent WGS cohort recapitulates the panel-seq findings and confirms predictive power of CHOP-like mutations. **A** Mutation frequencies of AML associated genes in diagnostic (D), complete molecular remission (CMR) and relapse samples (R). Mutations detected in at least two patients are depicted. **B** Clonal evolution analysis of *NPM1*^mut^ AML from diagnosis to CMR (*n* = 36 patients) of genes mutated in at least two patients. Lost mutations are depicted in blue, persistent mutations in gray and acquired mutations in orange. **C** Clonal evolution analysis of *NPM1*^mut^ AML from D to CMR and R (*n* = 8 patients) allows for higher temporal resolution and identifies three main patterns: mutations which could either persist at CMR and be lost at R or completely absent at both CMR and R (*ANAPC5, NRAS, FLT3*-TKD); CHIP-like mutations present at D, CMR and R (*TET2*, *IDH1*, *SRSF2*); mutations with oncogenic potential: gained at CMR and persistent at R or acquired de novo at R (*RAD21, TBC1D9, EZH2, EMC7, NACAD, RUNX1*); **D** Survival analysis of 36 patients from the WGS cohort stratified by clonal evolution patterns of novel defined CHIP-like mutations (*DNMT3A, TET2, ASXL1, SRSF2, IDH1, IDH2*): Kaplan–Meier plots depicting event-free survival (EFS, left panel) and overall survival (OS, right panel) of *NPM1*^mut^ AML patients based on the persistency/acquisition of CHIP vs CHOP like mutations at CMR. *P* values were calculated with the log-rank test.

For a comparison with the panel sequencing cohort, we focused our analysis on small variants found across the coding regions. At diagnosis, a total of 362 mutations were found across all 36 patients, including *NPM1* (10.1 mutations/patient, Fig. 5A). We observed mutations in several genes not known to be associated with AML, including: *ACOT8*, *ANAPC5*, *ANKFYI*, *CENPJ*, *COL14A1*, *ETNK1*, *GNAS*, *HAGHL*, and *ZNF622*.

At CMR, we detected a total of 138 mutations (3.8 mutations/patient, Fig. 5A). Again we observed mutations not previously associated with AML: *SETD1A*, *ARSD*, *ANAPC5*, *PLCG1*. Out of those all but 2 mutations in *ANAPC5* were not detected in the diagnostic sample.

In the eight patients with relapse, a total of 85 mutations was found (10.6 mutations/patient, Fig. 5A). The most common expected mutations were detected in: *IDH1*, *TET2*, *SRSF2*, *RAD21*, *RUNX1*. Unexpected mutations were detected in *EMC7* and *NACAD*.

The clonal evolution analysis of diagnostic and remission samples on all 36 patients identified a group of mutations which were completely or mostly lost and a second group which were almost exclusively acquired at CMR, indicating mutations with oncogenic potential. A third group showed a heterogeneous behavior that could identify CHIP-like mutations (Fig. 5B).

Focusing on the eight patients with available relapse samples, we identified three clonal evolution patterns: mutations mainly lost at CMR or relapse, mutations persistent at CMR or relapse (CHIP-like) and mutations gained at CMR and relapse (CHOP-like). This confirms the patterns found in the panel sequencing cohort (Fig. 5C).

Finally, we analyzed survival on this independent cohort using the above stratification based on persistency/acquisition of CHIP/CHOP-like mutations (Fig. 5D). Although this analysis was limited by the cohort size and the few events, we observed that patients with at least one persistent/acquired CHOP-like mutation at CMR showed a significantly poorer OS than those who did not (HR = 10.4, 1.2–86.6, *p* = 0.03).

## Discussion

In this report we selected a uniform cohort of 150 *NPM1*^mut^ AML patients all achieving CMR, and redefined the potential role of co-mutations persistent at remission. We identified the persistence of non DTA-mutations at CMR in a significant proportion of patients (15%), and confirm previous studies showing that the persistence of non-DTA-mutations in remission is detrimental [4, 6, 7]. However, those reports were focused on a variety of unselected AML. We have now addressed this phenomenon in the well-defined context of CMR in *NPM1*^mut^ AML. For this entity, MRD detection in clinical remission has long been established and is more informative for survival than the detection of co-mutations such as *FLT3*-ITD or *DNMT3A* [16, 19]. Our data on *NPM1*^mut^ AML in CMR suggest that the persistence of non-DTA mutations represents molecular residual disease. Furthermore, we show for the first time that also the acquisition of non-DTA mutations at CMR is an adverse prognostic factor in *NPM1*^mut^ AML.

The mutation diversity at CMR does not allow to reasonably address impact of single hits on survival even in our relatively large cohort, as we identified 24 different sub-cohorts according to persistent/acquired mutations. We therefore aimed to classify those mutations in favorable and adverse mutations. This is in line with recent efforts differentiating CHIP-like mutations from mutations with oncogenic potential [12, 20], termed CHOP-like mutations. Our analysis makes use of clonal hierarchy at diagnosis and the clonal evolution of co-mutations in CMR and relapse to classify mutations into those categories. We excluded patients with *NPM1* negative relapse to reduce the likelihood of secondary or t-AML [15]. The group of CHIP-associated mutations was extended to include mutations in *DNMT3A*, *TET2, IDH1*, *IDH2*, and *S*RSF2. All those mutations had a CHIP-like pattern in our analysis. This was further justified by the fact that also persistent/acquired *IDH1/2* or *SRSF2* mutations had no impact on survival along with mutations in *DNMT3A* and *TET2*. *ASXL1* mutations are a rare event in *NPM1*^mut^ AML and were not included in this group [16, 17]. We defined all other mutations (i.e., *FLT3*-TKD, *GATA2, NRAS*, *PTPN11, WT1, TP53, RUNX1*) as CHOP-like. Those were usually acquired at CMR and acquired/persistent at relapse. We prove a strong prognostic value of persistent and/or acquired CHOP-like mutations at CMR, in contrast to CHIP-like mutations. On the other hand, the detection of either CHIP or CHOP mutations at diagnosis did not have any impact on OS, highlighting the importance of sampling at CMR. Our data therefore allows the distinction of molecular residual disease from the persistence of a pre-malignant state which likely does not affect prognosis. We propose a model where CHIP-like mutations define a pre-malignant state in *NPM1*^mut^ AML, and the transformation to full AML is caused by the additional acquisition of driver mutations. This is backed by previous reports suggesting that *NPM1* mutation is a late event in leukemogenesis [15, 17, 21].

We confirm that the persistence of *DNMT3A*-R882 mutations is not associated with inferior survival [22]. However, contrasting earlier reports [23–25], also persistent *IDH*1/2 mutations were not associated with survival. We only identified eight patients with the exclusive persistence of *IDH* mutations, which did not show a dismal outcome. In other studies, adverse co-mutations accompanying *IDH1/2*, i.e., CHOP-like mutations, were not analyzed but could have been responsible for the inferior outcome.

We make use of a second cohort analyzed by WGS, focusing on the detection of small variants across the whole coding region. Albeit smaller, this cohort supports the definition of CHIP-like and CHOP-like mutations and the role of persistent/acquired CHOP-like mutations on outcome.

One could argue that different treatment strategies could perform better in eradicating molecular disease. In our analysis different inductions regimens did not have any impact on the distribution of CHIP/CHOP mutations at CMR.

CMR in *NPM1*^mut^ AML is an independent factor for good risk disease [16], however up to 30% of patients with CMR relapse. Here we provide a clinical tool where the detection of oncogenic mutations at CMR, acquired or persistent, is an independent prognostic factor facilitating early intervention in those patients. Studies with MRD guided therapy in AML show promising results: in the RELAZA2 trial, patients with MRD positive AML following conventional chemotherapy or allogeneic transplant were treated with azacytidine and showed a clinical meaningful benefit [26]. Based on the QUAZAR trial an oral formulation of azacytidine (CC-486) is the first approved maintenance therapy for AML [27], which could be especially worthy in patients with persistent CHOP-like mutations.

We also showed that patients relapsing with persistent or novel CHOP-like mutations have an inferior prognosis. Those patients represent an unmet clinical need and strategies like the RELAZA protocol, maintenance with demethylating substances [26, 28] or treatment with novel agents [29] could improve outcome.

In conclusion, our data show that even in the relatively favorable context of *NPM1*^mut^ AML following CMR, modern NGS based screening can identify patients at risk in order to develop personalized therapeutic strategies aimed at eradicating MRD and molecular residual disease to prevent relapse. The conduction of NGS-based MRD-guided clinical trials dedicated to this subset of *NPM1*^mut^ AML patients is highly warranted.

## Supplementary information

Supplemental Material

## Data Availability

Sequencing data was deposited at NCBI, accession number PRJNA745264.

## References

[1] DiNardo CD, Cortes JE (2016). Mutations in AML: prognostic and therapeutic implications. Hematol Am Soc Hematol Educ Program.

[2] Papaemmanuil E, Gerstung M, Bullinger L, Gaidzik VI, Paschka P, Roberts ND (2016). Genomic classification and prognosis in acute myeloid leukemia. N. Engl J Med.

[3] Ravandi F, Walter RB, Freeman SD (2018). Evaluating measurable residual disease in acute myeloid leukemia. Blood Adv.

[4] Jongen-Lavrencic M, Grob T, Hanekamp D, Kavelaars FG, Al HA, Zeilemaker A (2018). Molecular minimal residual disease in acute myeloid leukemia. N. Engl J Med.

[5] Klco JM, Miller CA, Griffith M, Petti A, Spencer DH, Ketkar-Kulkarni S (2015). Association between mutation clearance after induction therapy and outcomes in acute myeloid leukemia. JAMA.

[6] Rothenberg-Thurley M, Amler S, Goerlich D, Köhnke T, Konstandin NP, Schneider S (2018). Persistence of pre-leukemic clones during first remission and risk of relapse in acute myeloid leukemia. Leukemia.

[7] Morita K, Kantarjian HM, Wang F, Yan Y, Bueso-Ramos C, Sasaki K (2018). Clearance of somatic mutations at remission and the risk of relapse in acute myeloid leukemia. J Clin Oncol: Off J Am Soc Clin Oncol.

[8] Jaiswal S, Fontanillas P, Flannick J, Manning A, Grauman PV, Mar BG (2014). Age-related clonal hematopoiesis associated with adverse outcomes. N. Engl J Med.

[9] Genovese G, Kahler AK, Handsaker RE, Lindberg J, Rose SA, Bakhoum SF (2014). Clonal hematopoiesis and blood-cancer risk inferred from blood DNA sequence. N. Engl J Med.

[10] Steensma DP, Bejar R, Jaiswal S, Lindsley RC, Sekeres MA, Hasserjian RP (2015). Clonal hematopoiesis of indeterminate potential and its distinction from myelodysplastic syndromes. Blood.

[11] Abelson S, Collord G, Ng SWK, Weissbrod O, Mendelson Cohen N, Niemeyer E (2018). Prediction of acute myeloid leukaemia risk in healthy individuals. Nature.

[12] Valent P, Akin C, Arock M, Bock C, George TI, Galli SJ (2017). Proposed terminology and classification of pre-malignant neoplastic conditions: a consensus proposal. EBioMedicine.

[13] Parida L, Haferlach C, Rhrissorrakrai K, Utro F, Levovitz C, Kern W (2019). Dark-matter matters: discriminating subtle blood cancers using the darkest DNA. PLoS Comput Biol.

[14] Hollein A, Twardziok SO, Walter W, Hutter S, Baer C, Hernandez-Sanchez JM (2020). The combination of WGS and RNA-Seq is superior to conventional diagnostic tests in multiple myeloma: ready for prime time?. Cancer Genet.

[15] Hollein A, Meggendorfer M, Dicker F, Jeromin S, Nadarajah N, Kern W (2018). NPM1 mutated AML can relapse with wild-type NPM1: persistent clonal hematopoiesis can drive relapse. Blood Adv.

[16] Ivey A, Hills RK, Simpson MA, Jovanovic JV, Gilkes A, Grech A (2016). Assessment of minimal residual disease in standard-risk AML. N. Engl J Med.

[17] Cappelli LV, Meggendorfer M, Dicker F, Jeromin S, Hutter S, Kern W (2019). DNMT3A mutations are over-represented in young adults with NPM1 mutated AML and prompt a distinct co-mutational pattern. Leukemia.

[18] Acharya UH, Halpern AB, Wu QV, Voutsinas JM, Walter RB, Yun S (2018). Impact of region of diagnosis, ethnicity, age, and gender on survival in acute myeloid leukemia (AML). J Drug Assess.

[19] Schnittger S, Kern W, Tschulik C, Weiss T, Dicker F, Falini B (2009). Minimal residual disease levels assessed by NPM1 mutation-specific RQ-PCR provide important prognostic information in AML. Blood.

[20] Valent P, Kern W, Hoermann G, Milosevic Feenstra JD, Sotlar K, Pfeilstocker M, et al. Clonal hematopoiesis with oncogenic potential (CHOP): separation from CHIP and roads to AML. Int J Mol Sci. 2019;20:789. 10.3390/ijms20030789 PMC6387423 30759825

[21] Patel JL, Schumacher JA, Frizzell K, Sorrells S, Shen W, Clayton A (2017). Coexisting and cooperating mutations in NPM1-mutated acute myeloid leukemia. Leuk Res.

[22] Bhatnagar B, Eisfeld AK, Nicolet D, Mrozek K, Blachly JS, Orwick S (2016). Persistence of DNMT3A R882 mutations during remission does not adversely affect outcomes of patients with acute myeloid leukaemia. Br J Haematol.

[23] Ok CY, Loghavi S, Sui D, Wei P, Kanagal-Shamanna R, Yin CC (2019). Persistent IDH1/2 mutations in remission can predict relapse in patients with acute myeloid leukemia. Haematologica.

[24] Ferret Y, Boissel N, Helevaut N, Madic J, Nibourel O, Marceau-Renaut A (2018). Clinical relevance of IDH1/2 mutant allele burden during follow-up in acute myeloid leukemia. A study by the French ALFA group. Haematologica.

[25] Debarri H, Lebon D, Roumier C, Cheok M, Marceau-Renaut A, Nibourel O (2015). IDH1/2 but not DNMT3A mutations are suitable targets for minimal residual disease monitoring in acute myeloid leukemia patients: a study by the Acute Leukemia French Association. Oncotarget.

[26] Platzbecker U, Middeke JM, Sockel K, Herbst R, Wolf D, Baldus CD (2018). Measurable residual disease-guided treatment with azacitidine to prevent haematological relapse in patients with myelodysplastic syndrome and acute myeloid leukaemia (RELAZA2): an open-label, multicentre, phase 2 trial. Lancet Oncol.

[27] Wei AH, Döhner H, Pocock C, Montesinos P, Afanasyev B, Dombret H (2020). Oral azacitidine maintenance therapy for acute myeloid leukemia in first remission. N. Engl J Med.

[28] Huls G, Chitu DA, Havelange V, Jongen-Lavrencic M, van de Loosdrecht AA, Biemond BJ (2019). Azacitidine maintenance after intensive chemotherapy improves DFS in older AML patients. Blood.

[29] Cortes JE, Heidel FH, Hellmann A, Fiedler W, Smith BD, Robak T (2019). Randomized comparison of low dose cytarabine with or without glasdegib in patients with newly diagnosed acute myeloid leukemia or high-risk myelodysplastic syndrome. Leukemia.

