# Supplementary material for: Indeterminate and oncogenic potential: CHIP vs CHOP mutations in AML with NPM1 alteration
Source: Leukemia. 2021 Aug 10;36(2):394–402. doi: 10.1038/s41375-021-01368-1 (PMC8807394; doi:10.1038/s41375-021-01368-1)
Supplement: Supplementary file 1 — Supplemental Material [file 41375_2021_1368_MOESM1_ESM.pdf]

Supplemental Methods
Supplemental Tables: 6
Supplemental Figures: 7

Supplemental Methods

Genetic analyses

NGS panel of 63 genes associated with hematological malignancies:
ASXL1, ASXL2, ATM, BCL2, BCOR, BCORL1, BIRC3, BRAF, BTK, CALR, CBL, CSF3R, CSNK1A1, CXCR4, DNMT3A, EGR2, ETNK1, ETV6, EZH2, FBXW7, FLT3, FOXO1, GATA1, GATA2, ID3, IDH1, IDH2, JAK2, KIT, KLF2, KRAS, MAP2K1, MPL, MYC, MYD88, NF1, NFKBIE, NOTCH1, NOTCH2, NPM1, NRAS, PHF6, PIGA, PLCG2, POT1, PTPN11, RAD21, RUNX1, SAMHD1, SETBP1, SF3B1, SRSF2, STAG2, STAT3, STAT5B, TCF3, TET2, TP53, U2AF1, UBR5, WT1, XPO1, and *ZRSR2.*

Panel sequencing:
The library was generated with a TruSeq Custom Amplicon Low Input Kit (Illumina, San Diego, CA) following the manufacturer's protocol. The library was sequenced and demultiplexed on a Nextseq instrument (Illumina) as described previously[1]. The FASTQ files were further processed using Sequence Pilot software, version 4.4.0, Build 507 (JSI Medical Systems, Ettenheim, Germany), for alignment and variant calling. Validity of the somatic mutations was checked against the publicly accessible COSMIC v78 (http://cancer.sanger.ac.uk/cancergenome/projects/cosmic) and ClinVar databases. Functional interpretation was performed using SIFT 1.03 (http://sift.jcvi.org) and PolyPhen 2.0 (http://genetics.bwh.harvard.edu/pph2). Single- nucleotide polymorphisms were annotated according to the National Center for Biotechnology Information dbSNP (http://www.ncbi.nlm.nih. gov/snp; Build 147) and ExAC population frequency database[2]. Variants of uncertain significance were excluded from statistical analyses.

WGS sequencing:

WGS libraries were prepared with the TruSeq PCR free library prep kit and 150 bp paired-end sequences were generated on a NovaSeq 6000 or HiSeqX instrument with a median of 100x coverage (Illumina, San Diego, CA). The Illumina whole genome sequencing pipeline (version 5.0) and tumor-normal app (version 3.0) were used for variant calling of small nucleotide variants. In brief, WGS reads were mapped to human reference genome (GRCh37) using Isaac aligner (version 03.16.02.19).[3] Single nucleotide variants and small indels (SNVs) were called using Strelka (version 2.4.7).[4] The tumor-normal app used a mixture of genomic DNA from multiple anonymous donors (Promega, Madison, WI) as unmatched-normal controls.

We focused our analyses on the protein-coding regions of the genome. Variants were annotated using the Ensembl variant effect predictor[5], and only variants affecting protein-sequence or splice-acceptor/donor sites were retained. To remove potential germline variants, all SNVs with PASS flag were queried against the gnomAD database[6], and variants with global population frequencies >1% where excluded. We also took advantage of the multiplicity of samples per patient to filter out those variants bearing a VAF close to 50% or 100% across all timepoints, indicating either heterozygous or homozygous germline variants.

Statistical analyses

Differences in clinical, cytogenetic and molecular characteristics were tested using the Fisher's exact test for categorical variables and Mann-Whitney U test for continuous variables and when needed corrected for multiple comparisons using Bonferroni correction. Overall survival (OS), relapse-free survival (RFS) and event-free survival (EFS) were the selected endpoints to identify the outcome of patients bearing mutations at CMR or relapse, and were analysed using the log-rank test and the Cox proportional hazards model. OS, RFS and EFS were calculated from the sampling date until the date of the event of interest or censoring. EFS was defined from date of sampling to death, relapse, induction failure or censoring, whichever came first. OS after relapse was used as a secondary endpoint to account for the survival of patients based on their mutational landscape at relapse, and was calculated from the date of relapse until the date of death or censoring.

All statistical tests were two-sided and p-values <0.05 were considered statistically significant. The proportional hazards assumption was tested by interaction with time. SPSS software (version 19.0.0) (IBM Corporation, Armonk, NY, USA) and R software (version 3.6.3, employing the libraries survival and survminer) were used for statistical analysis.

	No relapse		Relapse		p-value
Death (no, %)	7/98	7%	16/52	31%	
5-years overall survival (OS) (%, range)	(global) 80% (72%-88%)				
	91%	(85%-98%)	63%	(50%-81%)	**0.0011**
5-years event-free survival (EFS) (%, range)	(global) 55% (46%-66%)				
OS-RFS (survival after relapse) (years - median, range)	/		1.3	(0.79-2.62)	/
Allogeneic transplant (no, %)	34/98	35%	27/52	52%	0.054
Allogeneic transplant after relapse (no, %)	/		21/24[*]	87.5%	/
Time to allogeneic transplant (TTT) (years - median, range)	(global) 0.79 (0.48-1.07)				
	0.42	(0.35-0.62)	1.38	(1.07-1.78)	<0.0001[§]

*: for 3/27 patients in the relapse group, allogeneic transplant date was not available

§: time to transplant is highly dependent on response to therapy. Patients who had a persisting clinical response and did not relapse were transplanted sooner than those who did not respond and relapsed.

ST1. Clinical endpoints of the whole *NPM1*^{mut} AML cohort. Number of deaths and patients undergoing allogeneic transplant, the medians of the 5-years overall survival (OS), 5-years event-free survival (EFS), survival after relapse and time to allogeneic transplant are provided. Data is stratified between patients with out relapse and patients experiencing clinical/molecular relapse. P-values are calculated using the Fisher’s exact test for categorical variables and the Kruskal-Wallis test for continuous ones.

	FLT3-ITD neg		*FLT3*-ITD pos		
	n	%	n	%	p-val
**DTA vs non-DTA**					
Persistency					
none	61	41%	26	17%	
DTA only	23	15%	17	11%	0.3699
non-DTA	14	9%	8	5%	
Acquisition					
none	82	55%	46	31%	
DTA only	10	7%	3	2%	0.5524
non-DTA	6	4%	2	1%	
**CHIP-like vs CHOP-like**					
Persistency					
none	61	41%	26	17%	
CHIP-like	36	24%	24	16%	0.399
CHOP-like	1	1%	1	1%	
Acquisition					
none	82	55%	46	31%	
CHIP-like	10	7%	3	2%	0.5524
CHOP-like	6	4%	2	1%	

ST2. Clonal evolution patterns of *NPM1*^{mut} AML cohort are not influenced by *FLT3*-ITD status at diagnosis. Number of patients with either persisting or acquired DTA/non-DTA or CHIP/CHOP-like mutations is provided. Patients are stratified by the absence or presence of *FLT3*-ITD mutations at diagnosis. The occurrence of different clonal evolution patterns does not depend on *FLT3*-ITD mutational status at diagnosis. P-values are calculated using the Fisher's exact test.

DTA vs non-DTA

	HR	lower-CI	upper-CI	p-val
**Overall survival**				
**Persistency**				
DTA only	1.19	0.41	3.49	0.7473
non-DTA	3.9	1.53	9.97	**0.0044**
**Acquisition**				
DTA only	1.69	0.49	5.79	0.398
non-DTA	2.81	0.81	9.74	0.103
**Persistency/acquisition**				
DTA only	1.45	0.54	3.89	0.463
non-DTA	3.61	1.32	9.82	**0.012**
**Event-free survival**				
**Persistency**				
DTA only	1.51	0.84	2.72	0.1702
non-DTA	2.25	1.19	4.27	**0.0126**
**Acquisition**				
DTA only	1.26	0.57	2.78	0.5699
non-DTA	3.05	1.29	7.19	**0.0107**
**Persistency/acquisition**				
DTA only	1.54	0.87	2.73	0.1359
non-DTA	2.69	1.39	5.18	**0.003**

ST3. Hazard risks of *NPM1*^{mut} AML patients stratified by clonal evolution patterns based on DTA/non-DTA mutations. Hazard risks (HR) and their related confidence intervals (CI) and p-values (p-val) are provided based on the clonal evolution patterns of patients with either persisting or acquired DTA/non-DTA mutations. HR were calculated using Mantel-Cox Univariate analysis.

		HR (95% CI, p-value)
**DTA vs non-DTA**	None	–
	DTA persisting/acquired	1.54 (0.43–5.46, p=0.504)
	non–DTA persisting/acquired	3.76 (1.01–13.97, p=0.047)
**Allo-transplant**	No	–
	Yes	0.24 (0.07–0.78, p=0.017)
**Karyotype**	Normal	–
	Aberrant	0.68 (0.14–3.25, p=0.627)
**Gender**	Female	–
	Male	1.58 (0.63–3.95, p=0.330)
**Age**	<60	–
	>=60	0.75 (0.26–2.20, p=0.605)
***FLT3*-ITD**	Non mutated	–
	Mutated	0.72 (0.26–1.97, p=0.523)
DNMT3A	Non mutated	–
	Mutated	2.06 (0.73–5.77, p=0.169)

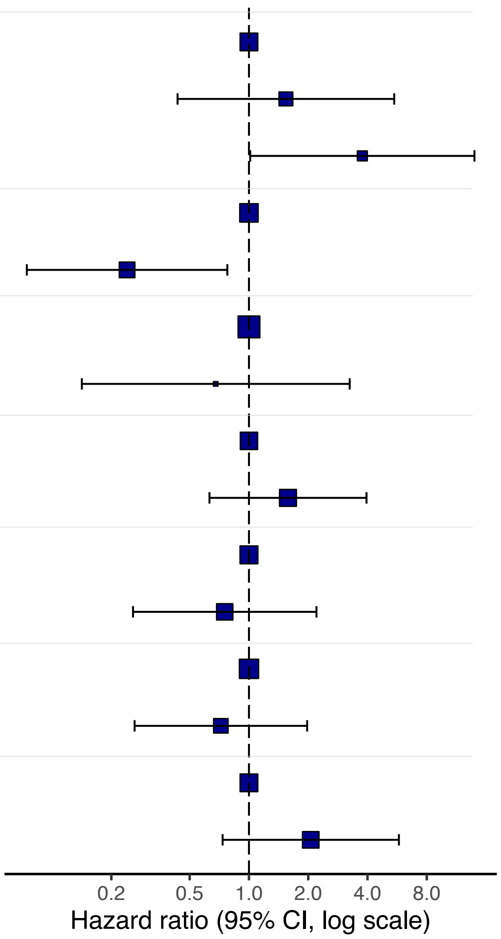

ST4. Cox proportional hazards multivariate model incorporating clonal evolution patterns by presence/absence of non-DTA mutations at CMR, clinical/molecular risk factors and allogeneic hematopoietic stem-cell transplantation. The persistency/acquisition of non-DTA hits at CMR is an independent predictor of outcome in *NPM1*^{mut} AML patients. Hazard ratio (HR) at 95% confidence interval and p-values for each variable are given.

CHIP-like vs CHOP-like

	HR	lower-CI	upper-CI	p-val
**Overall survival**				
**Persistency**				
CHIP-like	1.78	0.75	4.21	0.188
CHOP-like	30.28	6.06	151.2	**<0.0001**
**Acquisition**				
CHIP-like	1.69	0.49	5.79	0.398
CHOP-like	2.81	0.81	9.74	0.103
**Persistency/acquisition**				
CHIP-like	1.53	0.61	3.87	0.3647
CHOP-like	5.54	1.82	16.86	**0.0025**
**Event-free survival**				
**Persistency**				
CHIP-like	1.67	1.01	2.78	0.0472
CHOP-like	8.69	2.1	38.25	**0.0031**
**Acquisition**				
CHIP-like	1.26	0.57	2.78	0.5699
CHOP-like	3.05	1.29	7.2	**0.0107**
**Persistency/acquisition**				
CHIP-like	1.58	0.92	2.71	0.0932
CHOP-like	4.5	2.01	10.06	**0.0002**

ST5. Hazard risks of *NPM1*^{mut} AML patients stratified by clonal evolution patterns based on CHIP/CHOP-like mutations. Hazard risks (HR) and their related confidence intervals (CI) and p-values (p-val) are provided based on the clonal evolution patterns of patients with either persisting or acquired CHIP/CHOP-like mutations. HR were calculated using Mantel-Cox Univariate analysis

	n	%
**Gender**		
Male	18	50%
Female	18	50%
**WHO AML subtype**		
AML with minimal differentiation	0	0%
AML without maturation	15	42%
AML with maturation	8	22%
Acute myelomonocytic leukemia	9	25%
Acute monoblastic/monocytic leukemia	3	8%
Pure erythroid leukemia	1	3%
NA	0	0%
**Karyotype**		
Normal	34	94%
Aberrant*	2	6%
	median	range
**Age**	56.3	28.3-79.8
**Hb**	9.1	4-16
**Thrombocytes ($x10^3$)**	70	10-279
**Leucocytes ($x10^3$)**	31	1-189

ST6. Population table of the WGS cohort of 36 *NPM1*^{mut} AML patients.

Supplemental figure 1 Cappelli et al.

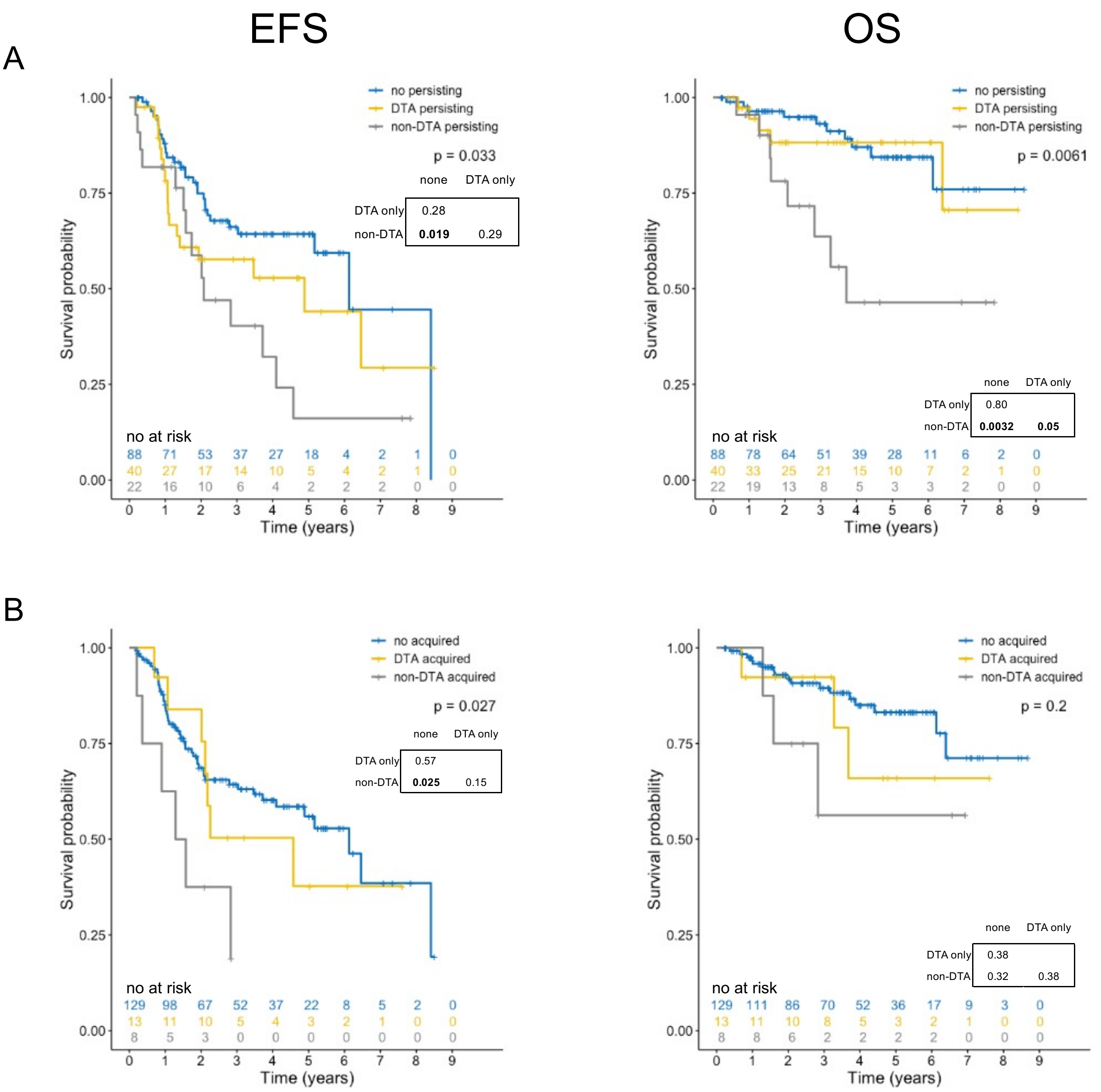

S1. Persisting/acquired non-DTA mutations at CMR confer inferior survival in AML with mutated *NPM1*. (A, B) Survival analysis of patients with *NPM1*^{mut} AML stratified by clonal evolution patterns of DTA vs non-DTA mutations. Kaplan-Meier plots depicting event-free survival (EFS left panel) and overall survival (OS right panel) of *NPM1*^{mut} AML patients based on the persistency (A) or the acquisition (B) of non-DTA mutations at CMR. Patients with detectable non-DTA mutations at CMR have a worse prognosis than those who do not. P-values were calculated with the log-rank test.

A

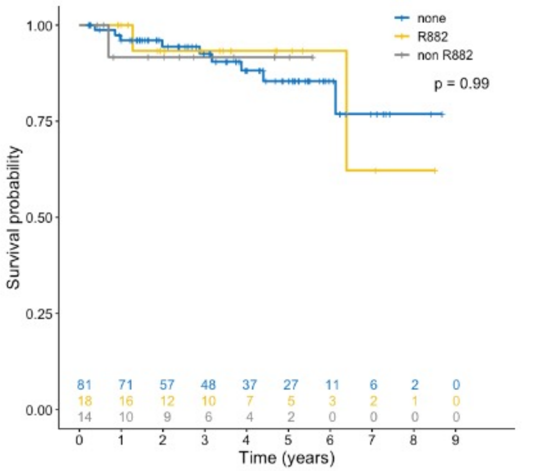

B

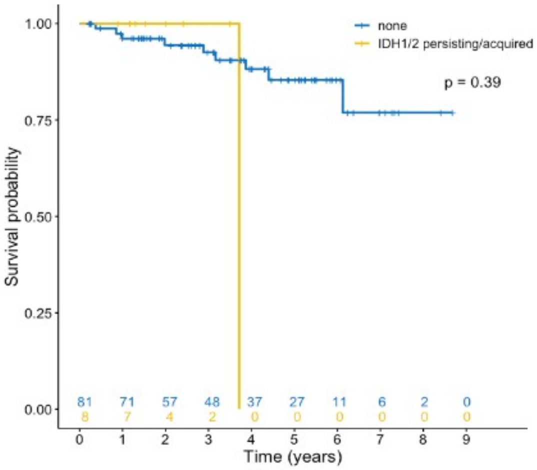

C

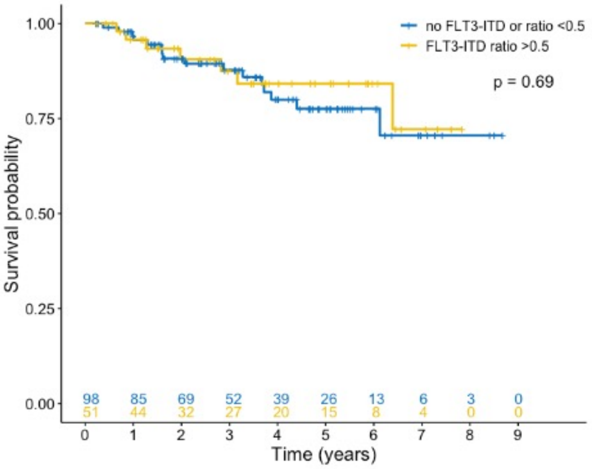

S2. Persistence of *DNMT3A (R882 or non-R882)* and *IDH1/2* at CMR or ELN risk group at diagnosis does not influence sruvival. (A, B, C) Survival analysis of patients with *NPM1*^{mut} AML stratified by persistence of *DNMT3A (R882 or other)* and *IDH1/2* at CMR and ELN risk group at diagnosis (*FLT3*-ITD ratio > or < 0.5). Kaplan-Meier plots depicting overall survival (OS) of *NPM1*^{mut} AML patients based on the persistency/acquisition of *DNMT3A*-R882/non-R882 (A) or IDH1/2 (B) mutations at CMR. Patients with no detectable mutations at CMR are used as comparator. (C) Kaplan-Meier plot depicting OS of patients stratified by absence or presence of *FLT3*-ITD (below or above the 0.5 threshold) at diagnosis. P-values were calculated with the log-rank test.

A

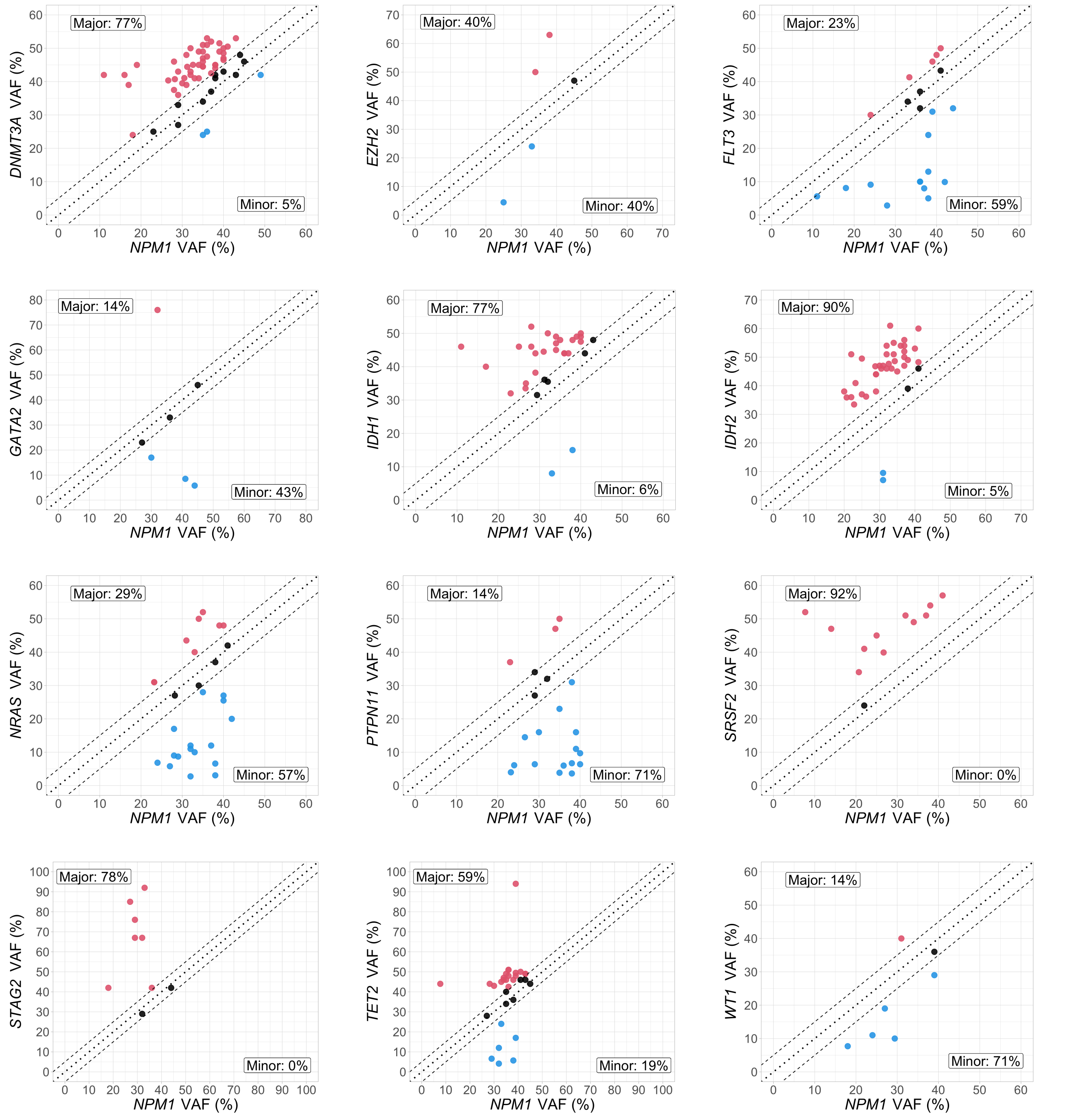

S3A. Clonal hierarchy of co-mutations in *NPM1*^{mut} AML at diagnosis. Scatterplots depicting the VAF of *NPM1* vs other co-mutations at diagnosis. Only co-mutations present in $\geq$5 patients are included. “Major” refers to the percentage of co-mutations with a higher VAF than *NPM1* (red dots), “minor” refers to the percentage of co-mutations with a lower VAF than *NPM1* (blue dots). The two dashed lines represent the VAF threshold to define major or minor hits ($\pm$5%). Black dots represent co-mutations with a VAF similar to *NPM1* (less than $\pm$5%).

B

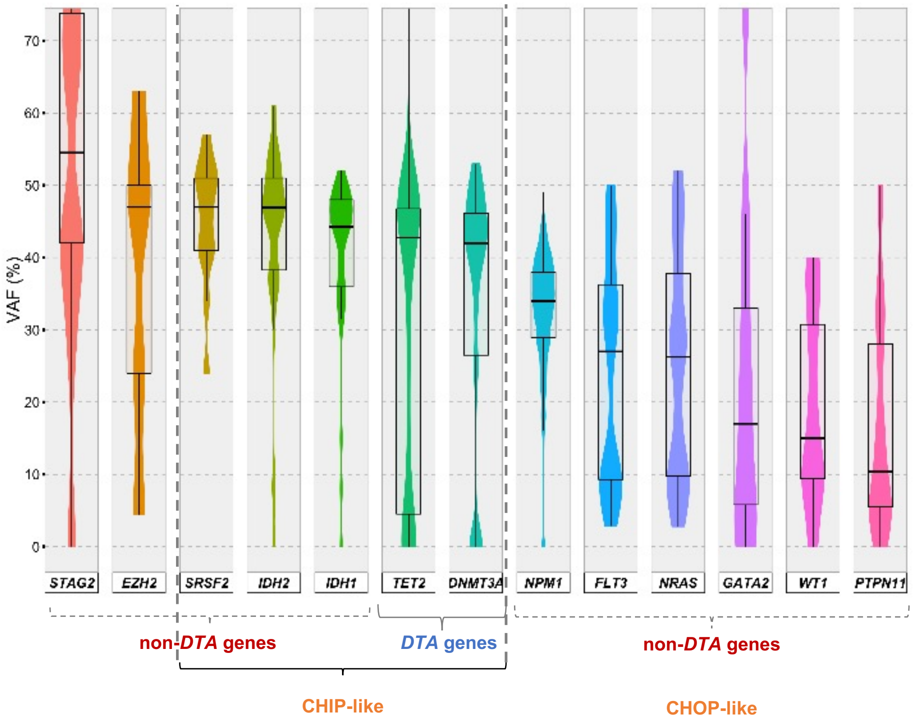

S3B. Clonal hierarchy of co-mutations in *NPM1*^{mut} AML at diagnosis. Variant allele frequency (VAF) distributions of *NPM1* and co-mutations, ordered by medians. Genes mutated in at least 5 patients were included. Color codes are based on clonal evolution analysis indicating non-DTA genes (red), DTA genes (blue). *NPM1* was a second hit mutation compared to: *STAG2* (100% of cases), *EZH2* (100%), *DNMT3A* (77%), *IDH1* (77%), *IDH2* (90%), *SRSF2* (92%) and *TET2* (59%). On the other hand, we identified a number of mutations with a generally lower VAF compared to *NPM1*. Those were mutations in *FLT3*-TKD (59% of the cases), *GATA2* (43%), *NRAS* (57%), *PTPN11* (71%) and *WT1* (71%). This clonal hierarchy suggests that some muations behave as CHIP-like and others as CHOP-like mutations in the context of *NPM1*^{mut} AML.

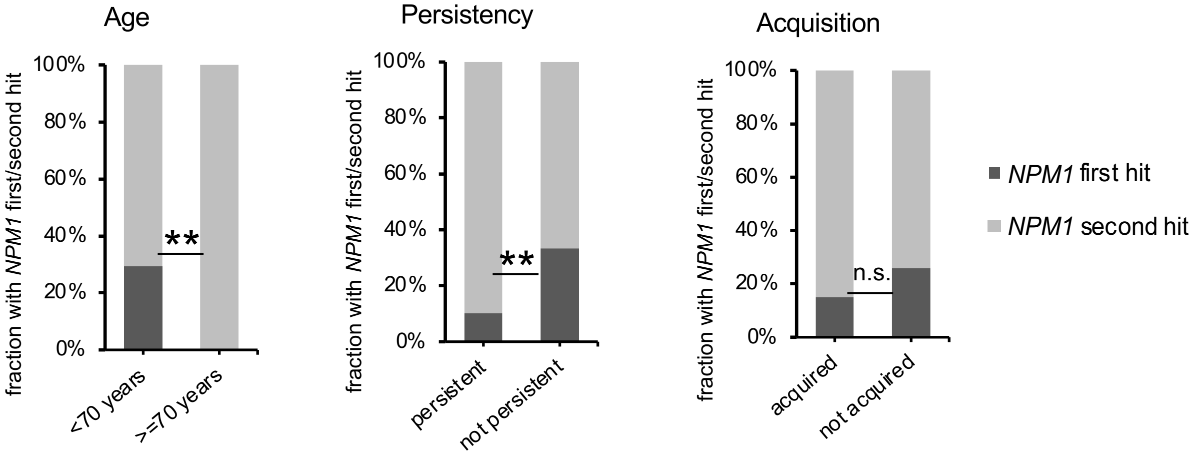

S4. Clonal hierarchy is associated with age and clonal evolution. Shown is age, rate of persistent or acquired mutations at complete molecular remission (CMR) in relation to *NPM1* defined as first or second hit mutation. Patients carrying *NPM1* as second hit are usually older (>70 years old) and show a higher rate of persisting or acquired mutations at CMR. P-values were calculated with Fisher's exact test (*=p<0.05, **=p<0.01, ***=p<0.0001).

Supplemental figure 5

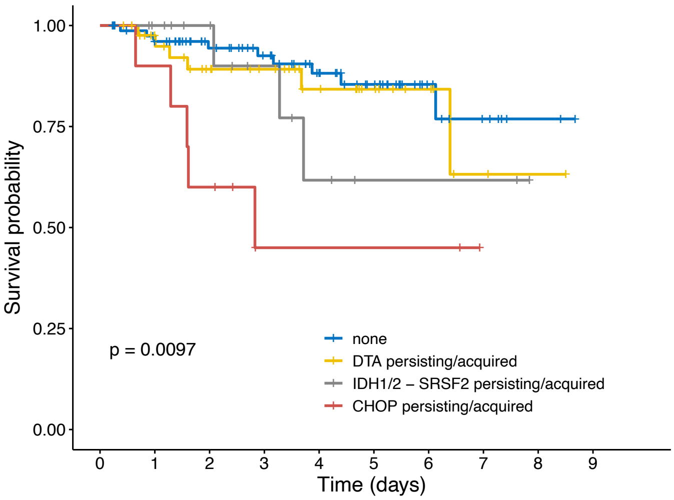

	0	1	2	3	4	5	6	7	8	9
	81	71	57	48	37	27	11	6	2	0
	43	36	28	23	16	10	6	2	1	0
	16	14	11	7	4	2	2	2	0	0
	10	9	6	2	2	2	2	0	0	0

Time (days)

	none	DTA persistent/acquired	IDH1/2 - SRSF2 persisting/acquired
DTA persistent/acquired	0.56		
IDH1/2 - SRSF2 persisting/acquired	0.26	0.65	
CHOP persisting/acquired	**0.0006**	**0.028**	0.11

S5. Presence of *DTA, IDH1/2 and SRSF2* mutations (CHIP mutations) at CMR does not influence survival. Kaplan-Meier plot depicting overall survival (OS) of patients with *NPM1*^{mut} AML stratified by persistence/acquisition of *DTA (DNMT3A, TET2), IDH1/2* and *SRSF2* or CHOP mutations at CMR. Patients with no detectable mutations at CMR are used as comparator. The detection of CHOP mutations at CMR is an independent negative prognostic factor, regardless of CHIP mutations. P-values were calculated with the log-rank test.

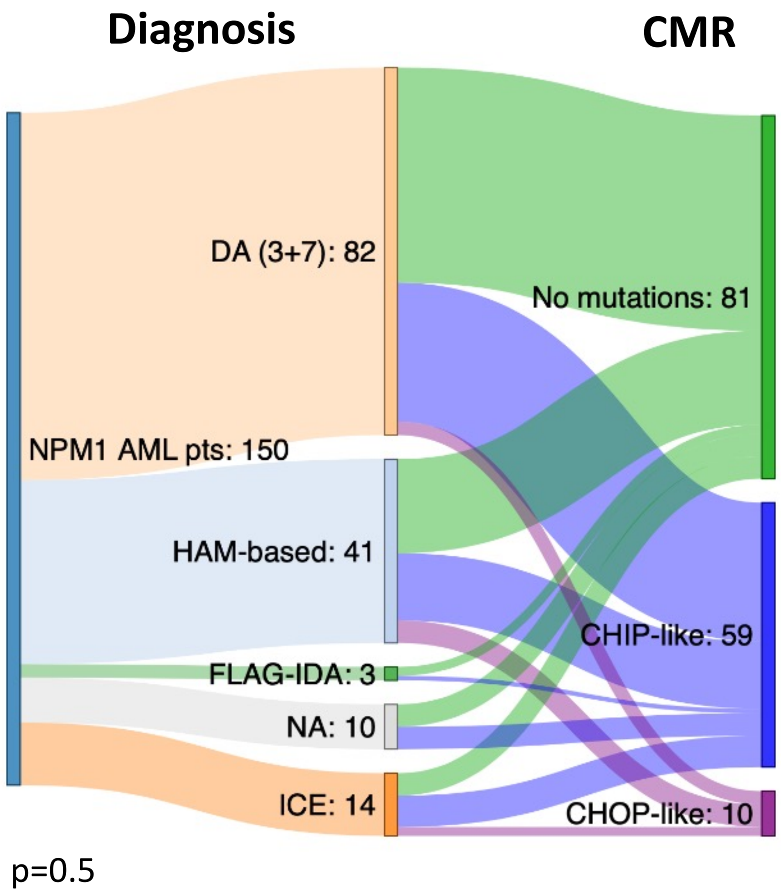

S6. Different chemotherapeutic schemes do not influence acquisition/persistence of CHIP/CHOP hits at CMR. Sankey plot depicting the distribution of the 150 *NPM1*^{mut} AML patients based on therapeutic induction/consolidation regimens received after diagnosis (left) and the prevalence of CHIP/CHOP mutations at complete molecular remission (CMR) (right). No bias in the distribution of mutations based on therapeutic regimen was observed (p=0.5). DA(3+7): ; HAM-based: ; FLAG-IDA: ; ICE: : NA: not available.

Mutational status at diagnosis

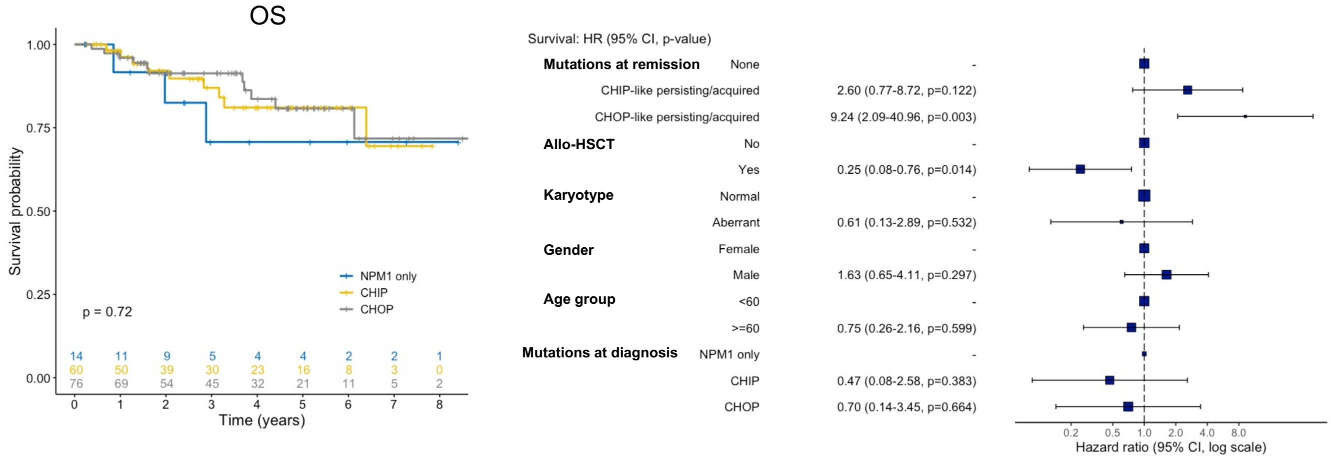

S7. Presence of either CHIP or CHOP mutations at diagnosis does not influence survival. (Left) Kaplan-Meier plot depicting overall survival (OS) of patients with *NPM1*^{mut} AML stratified by persistence/acquisition of *DTA (DNMT3A, TET2), IDH1/2* and *SRSF2* or CHOP mutations at CMR. Patients with only *NPM1* detectable are used as comparator. The detection of CHIP/CHOP mutations at diagnosis does not influence survival. P-values were calculated with the log-rank test. (Right) Cox proportional hazards multivariate model incorporating presence/absence of CHIP/CHOP mutations at diagnosis vs CMR, clinical/molecular risk factors and allogeneic hematopoietic stem-cell transplantation. The presence of CHOP hits is an independent predictor of outcome in *NPM1*^{mut} AML patients only when detected at CMR but not at diagnosis. Hazard ratio (HR) at 95% confidence interval and p-values for each variable are given.

References

1. Delic S, Rose D, Kern W, et al. Application of an NGS-based 28-gene panel in myeloproliferative neoplasms reveals distinct mutation patterns in essential thrombocythaemia, primary myelofibrosis and polycythaemia vera. *Br J Haematol*. 2016;175(3):419-426.
2. Lek M, Karczewski KJ, Minikel EV, et al. Analysis of protein-coding genetic variation in 60,706 humans. *Nature*. 2016;536(7616):285-291.
3. Raczy C, Petrovski R, Saunders CT, et al. Isaac: ultra-fast whole-genome secondary analysis on Illumina sequencing platforms. *Bioinformatics*. 2013;29(16):2041-2043.
4. Kim S, Scheffler K, Halpern AL, et al. Strelka2: fast and accurate calling of germline and somatic variants. *Nat Methods*. 2018;15(8):591-594.
5. McLaren W, Gil L, Hunt SE, et al. The Ensembl Variant Effect Predictor. *Genome Biol*. 2016;17(1):122.
6. Karczewski KJ, Francioli LC, Tiao G, et al. The mutational constraint spectrum quantified from variation in 141,456 humans. *Nature*. 2020;581(7809):434-443.
